# Pertussis: Challenges Today and for the Future

**DOI:** 10.1371/journal.ppat.1003418

**Published:** 2013-07-25

**Authors:** James D. Cherry

**Affiliations:** 1 Pediatric Infectious Diseases, Mattel Children's Hospital, Los Angeles, California, United States of America; 2 The Department of Pediatrics, David Geffen School of Medicine at the University of California Los Angeles, Los Angeles, California, United States of America; Duke University Medical Center, United States of America

## What Is Pertussis?

Pertussis is a truly unique contagious respiratory disease [1]–[4]. It is mainly caused by *Bordetella pertussis*, but similar cough illnesses can be caused by *B. parapertussis* in young children and *B. holmesii* in adolescents and adults. (The remainder of this communication will be restricted to *B. pertussis* infection and illness.)

Illness due to *B. pertussis* has a number of characteristics that differentiate it from all other severe respiratory illnesses [1]–[4]. The basic illness is noninflammatory in nature and occurs without significant fever. Mortality is greater in girls than in boys. The characteristic, nonproductive paroxysmal cough followed by periods of total respiratory normalcy is different from all other infectious cough illnesses. Concomitant respiratory viral infections and secondary bacterial infections that include fever and other manifestations of inflammation have over the years led to confusion as to the fundamental illness caused by *B. pertussis*.

Neither *B. pertussis* infection or pertussis vaccination elicit long-lasting immunity [1], [4]. Infection and illness repeatedly occur in all ages throughout life.

In the prevaccine era, pertussis morbidity and mortality was staggering. Vaccines (killed whole organisms—DTwP) were developed in the 1930s, and their subsequent use beginning in the late 1940s resulted in a 157-fold decrease in the pertussis attack rate [1], [4]. Because DTwP vaccines contained endotoxin, their use was associated with considerable side effects. Technology of the 1970s and 1980s enabled the production of acellular vaccines (DTaP) that were less reactogenic because of the removal of endotoxin. These DTaP vaccines only contained a small number of antigens in comparison with DTwP vaccines. DTaP vaccines were put into universal use in the United States in the late 1990s.

## Misconceptions about *B. pertussis* Infection and Disease

**Figure 1:**
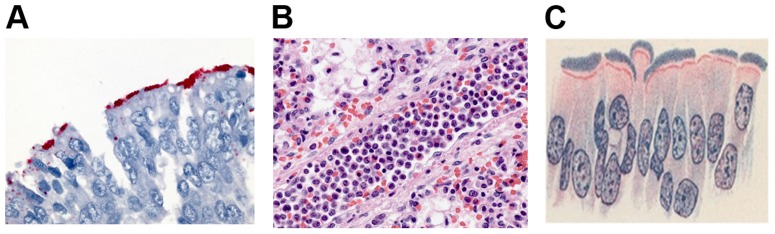
**A: *Bordetella pertussis* on Ciliated Cells of Bronchus.** Immunohistochemical localization of *B. pertussis* bacteria in the cilia of respiratory epithelium lining a bronchus of an infant who died from pertussis. Image courtesy of Christopher Paddock, M.D., Centers for Disease Control and Prevention. **B: Aggregates of Leukocytes in a Pulmonary Arteriole in Pertussis.** Pulmonary arteriole from an infant with fatal pertussis, showing intravascular aggregates of mixed leukocytes, comprising predominantly mature and band neutrophils, eosinophils, and monocytes. Image courtesy of Christopher Paddock, M.D., Centers for Disease Control and Prevention. **C: Drawing of *Bordetella pertussis* on Ciliated Cells in the Trachea.** Published over 100 Years Ago. Ciliated epithelium lining trachea of child dying in acute stage of whooping cough. Large numbers of minute bacilli present between the cilia. x1,000 [27].

Pertussis due to *B. pertussis* infection is exclusively a human disease [4]. However, over the last hundred years the microbiologic characteristics of *B. pertussis* have largely been determined using numerous animal-model and organ-culture systems [5]. In my opinion, the use of these systems has led to many prevailing views that are wrong. Human infections caused by *B. pertussis* are due to a number of proteins that are adhesins or that interfere with innate immunity [1], [4], [5]. Available evidence (restricted to pure *B. pertussis* infections without concomitant viral or secondary bacterial infections) suggests that clinical illness is noninflammatory in nature (Figure 1A and 1C) and that it is due to two toxins [5]–[7]. One toxin is pertussis toxin (PT), which is well characterized, and the second toxin (“cough toxin”) has not as yet been discovered [5].

PT in some young infants causes extreme leukocytosis with lymphocytosis and is associated with pulmonary hypertension, pneumonia, and death [6], [7] (Figure 1B). Once a person has been immunized or has had *B. pertussis* infection, the manifestations of PT are not seen again in older children or adults. However, cough illness due to *B. pertussis* infection occurs in persons of all ages, supporting the concept of the as yet to be identified “cough toxin.”

## Why Has There Been a Resurgence of Pertussis?

In the United States, there has been a general increase in reported pertussis, which began 30 years ago [8]–[11]. The characteristics of this resurgence changed dramatically in 2005, and the greatest number of reported cases since ∼1960 occurred in 2012. There are five possible reasons for the resurgence: 1) genetic changes in *B. pertussis*; 2) a decrease in vaccine efficacy; 3) a more rapid occurrence of waning immunity; 4) increased recognition and reporting of pertussis; and 5) newer laboratory diagnostic tests.

It is my opinion that the initial observations of more pertussis in the 1980s and 1990s were due to a general increased awareness about pertussis because of hundreds of publications relating to the development and study of acellular pertussis vaccines. The increased awareness of adolescent and adult pertussis was fostered by the use of single serum serologic study in numerous investigations and the routine use of this method in Massachusetts since 1991 [1], [4], [8]. The use of PCR has been a significant factor in finding more cases over the last four years.

The remaining three possible causes of resurgence noted above will be discussed in the next section.

## Key Reasons for Vaccine Failure

There is clear evidence that all DTaP vaccines have less efficacy than good DTwP vaccines [12], [13]. Furthermore, the stated efficacy of all vaccines has been inflated by the use of the WHO case definition, which was described in 1991 [14]. This definition required laboratory confirmation and ≥21 days of paroxysmal cough [14]. In addition, all efficacy has also been inflated by “observer bias” [12], [13], [15]. It is likely that the efficacy of the DTaP vaccines in use in the United States today is less than 70% [12], [13], [15].

In the period 1985 to 1995, nine pertussis vaccine efficacy trials were carried out, but unfortunately only two of these trials were performed in a manner that would identify serologic correlates of immunity [1], [4], [16]–[18]. In both correlates of immunity studies, antibody to pertactin (PRN) and fimbriae (FIM) correlated with protection against disease in ∼70% of children when exposed in the household. In addition, in both studies antibody to PT correlated only modestly with protection, and antibody to filamentous hemagglutinin (FHA) did not have any correlation with protection. Finally, in both trials antibody to PT had an adverse effect on correlates of protection induced by antibody to FIM.

One frequently overlooked fact of the vaccine trials two decades ago is that acellular vaccine efficacy was directly related to the number of antigens that a vaccine contained [1], [4], [12], [13]. Specifically, a 5-component vaccine (PT, FHA, PRN, FIM 2/3) was better than a 3-component vaccine (PT, FHA, PRN); 3-component vaccines were better than 2-component vaccines (PT, FHA); and 2-component vaccines were better than 1-component vaccines (PT) [1], [12], [13]. The apparent greater efficacy of the PT/FHA vaccines as compared to PT vaccines may not be due to the addition of FHA, but due to the fact that the PT/FHA vaccines at that time also contained PRN and perhaps FIM [1].

The universal use of pertussis vaccines has been associated with genetic changes in circulating *B. pertussis* strains [11], [12], [19], [20]. This was noted ∼50 years ago when it was found that only one of 11 DTwP vaccines in England contained agglutinogen 3, whereas all contained agglutinogen 2 [19]. At that time, agglutinogen 2–containing strains were recovered from only non-vaccinated children with pertussis, whereas agglutinogen 3 strains were recovered from children with pertussis who were vaccine failures.

Since DTwP vaccines contain many additional “protective” antigens in addition to PT, FHA, PRN, and FIM 2/3, genetic change was apparently not a problem in the DTwP era. However, today with DTaP vaccines, genetic change should be a major concern regarding vaccine efficacy.

It has been suggested that an increase in the correlation of *B. pertussis* strains containing the *ptx* P3 allele rather than the *ptx* P1 allele has led to vaccine failures and more severe illness [11], [12], [21]. However, there is no convincing evidence supporting this suggestion. Recently, the circulation of PRN-deficient mutants has been reported [22]. Since it was shown in the serologic correlations of immunity studies [16], [17] that antibody to PRN correlated with protection ∼70% of the time, it would seem possible that this deficiency could lead to increased vaccine failures in the United States if the deficient mutants became widespread.

Also alarming is the dramatic increase in strains with *fim* 3B in the present DTaP vaccine era in the U.S. [20]. This could lead to a decrease in efficacy of the U.S. vaccines, which contain FIM 2/3 as well as PRN. However, demonstrating increased vaccine failure with either the PT, FHA, PRN, or the PT, FHA, PRN, FIM 2/3 vaccines will be difficult because antibody to the B subunit of PT provides considerable efficacy against typical pertussis as demonstrated in Denmark, where a PT toxoid vaccine has been used for ∼15 years [11], [12].

Less clear but also important is the balance of antigens in vaccines [11], [12], [16], [17], [23]. For example, the antibody response patterns from two good DTwP vaccines to PT, filamentous hemagglutinin (FHA), and FIM are different than those in children who have received acellular pertussis vaccines [24]. The whole-cell vaccines produced low levels of antibody to PT and FHA and high levels of antibody to PRN and FIM [24]. In contrast, the acellular vaccines both produced high levels of antibody to PT and FHA. Previous studies suggested antagonism between PT and FIM in regard to vaccine efficacy [16], [17], and a study by Weiss et al. [23] suggested antagonism between PT and PRN. Also, since in two studies antibody to FHA did not correlate with vaccine efficacy, it is possible that antibody to this antigen is also antagonistic to the effects of PRN and FIM [16], [17].

Another factor that could contribute to DTaP vaccine failure as compared to whole vaccines is linked epitope suppression [18]. Our group previously found that in acellular vaccine failures the antibody response to vaccine antigens was brisk, whereas it was severely suppressed in regard to non-vaccine antigens. I thought that this might not be particularly important in the big picture, since it would be likely that persons would be exposed to PRN and FHA in an unlinked fashion when infected with other *Bordetella* species. However, two recent studies suggest that linked epitope suppression may be very important [25], [26]. Specifically, in both Brisbane, Australia and in Oregon it has been found that persons whose initial priming was with a whole-cell vaccine (just one dose) had lower pertussis attack rates than those primed with acellular vaccines when later exposed.

## The Future

Recently, much attention has been devoted to the resurgence of pertussis and solutions to the problem. On the table are new acellular pertussis vaccines and live attenuated vaccines. However, as I have stated previously, from the data presented above, the development of DTaP vaccines that are better than DTwP vaccines would seem unlikely [11], [12]. And, although not discussed here, it is my opinion that live vaccines will not provide as good protection as good DTwP vaccines [12]. Therefore, I believe a high priority should be given to the development of less reactogenic whole-cell vaccines.
